# Treatment of pacemaker-induced superior vena cava syndrome by direct oral anticoagulant

**DOI:** 10.1186/s12959-023-00459-6

**Published:** 2023-02-13

**Authors:** Weijun Huang, Yingwen Chen, Ziwei Liu, Yuli Huang, Yunzhao Hu

**Affiliations:** 1 Department of Cardiology, Shunde Hospital, Southern Medical University (The First People’s Hospital of Shunde), Shunde, Foshan, China; 2 Department of Radiology, Shunde Hospital, Southern Medical University (The First People’s Hospital of Shunde), Shunde, Foshan, China

**Keywords:** Superior vena cava syndrome, Pacemaker, Direct oral anticoagulants, Thrombosis

## Abstract

**Background:**

The use of cardiac implantable electronic devices has grown substantially over the past two decades, lead-related vascular issues are commonly encountered in clinical practice. Superior vena cava (SVC) syndrome due to pacemaker leads is an uncommon complication. Anticoagulation remains the mainstay of therapy to restore some degree of patency and relieve swelling. However, there are limited clinical trials on direct oral anticoagulants (DOACs).

**Case presentation:**

We report a case of an 80-year-old man who developed SVC syndrome after transvenous pacemaker implantation with symptoms of obstruction that were significantly relieved after four months of DOACs. His symptoms had completely resolved nine months later.

**Conclusions:**

DOACs are effective in the treatment of SVC syndrome after pacemaker implantation, representing an important new approach. It is a very good choice for patients who do not want to undergo interventional therapy.

## Background

Superior vena cava (SVC) syndrome due to pacemaker leads is a distinct diagnostic rarity. Most patients are asymptomatic because of the progressive formation of collateral veins. However, in recent decades, thrombotic occlusion of SVC is frequently encountered because of the presence of central venous catheters and pacemaker leads [1, 2]. Treatment options for lead-related venous occlusion are mainly supported by case series and anecdotal experience [2]. We report a case of an 80-year-old man who presented with SVC syndrome after transvenous pacemaker implantation with symptoms relieved after 4 months of direct oral anticoagulants (DOACs).

## Case presentation

**Fig. 1:**
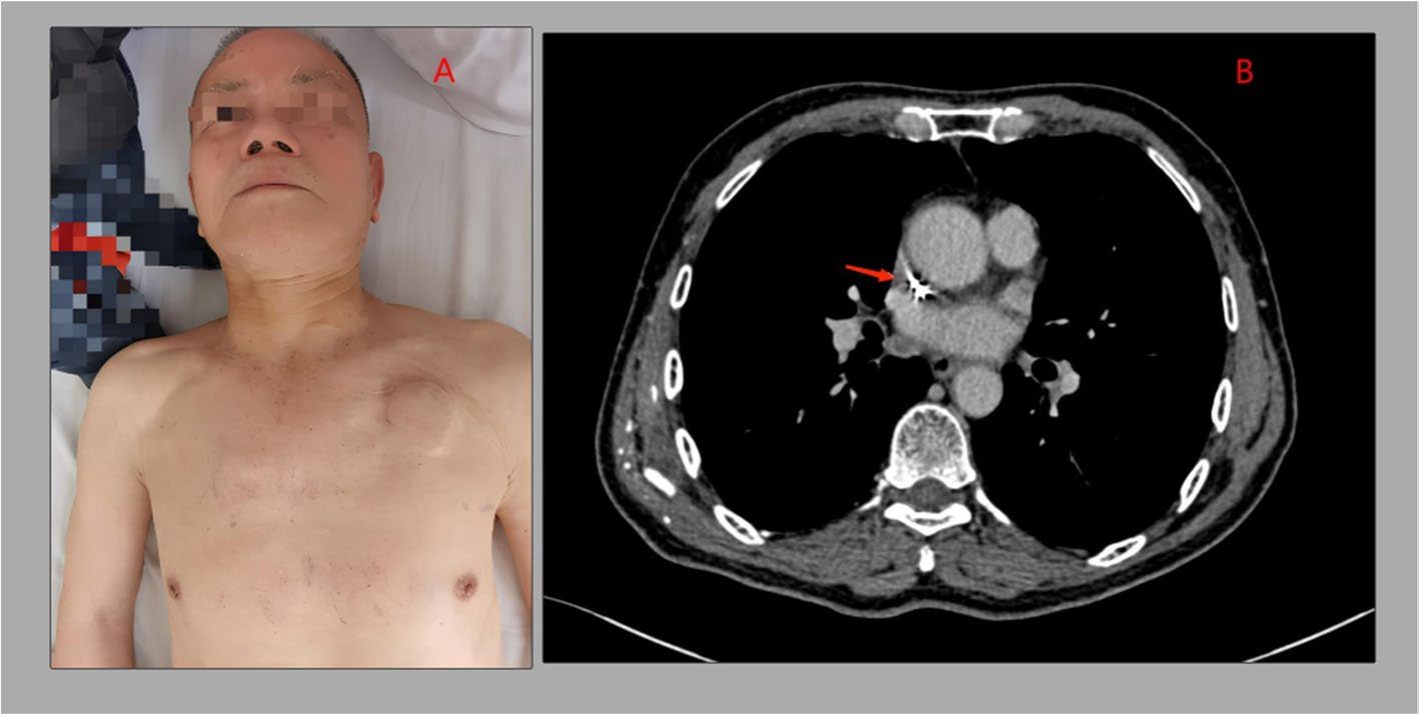
The image show edema and prominent engorged vasculature in the face, neck, and anterior chest wall (**A**). Transverse plane image of contrast-enhanced CT scan demonstrating a thrombus obstructing the SVC around indwelling pacemaker leads(arrow) (**B**)

An 80-year-old man was admitted to our cardiovascular clinic because he developed facial edema, and his neck gradually thickened and bleeding from the left eye. His medical history was notable for second-degree atrioventricular blocks: Mobitz type II, for which he had undergone placement of a pacemaker about 2 years earlier. In addition, the patient was initially concerned about the risk of bleeding and received only rivaroxaban 10 mg once daily for paroxysmal atrial fibrillation. There was no history of fever, haemoptysis, hoarseness of voice, and weight loss. Physical examination was notable for obvious varicosities across the anterior chest, subcutaneous tissue edema was seen on both chest walls as well as myofascial planes of the neck and face. (Fig. 1: A). SVC syndrome was initially diagnosed and further investigation was conducted to determine its etiology. Complete blood count, coagulation, renal and hepatic function were within normal limits.

**Fig. 2:**
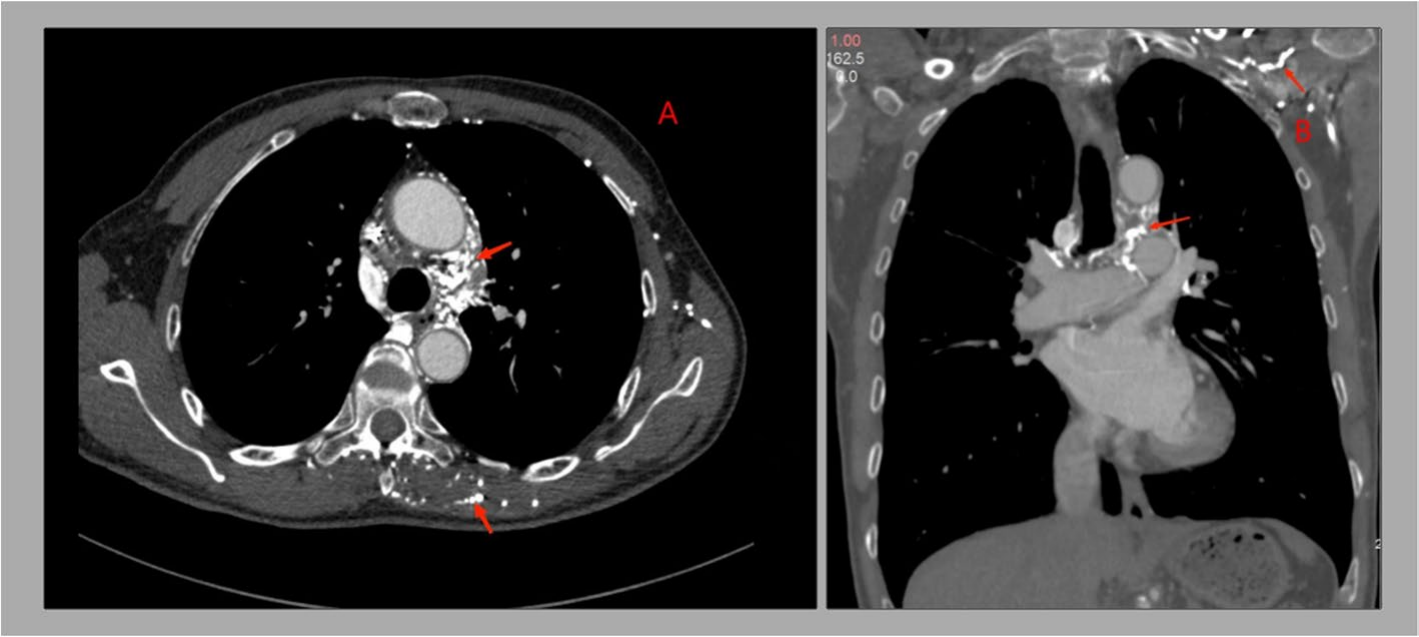
The image of contrast-enhanced CT scan showing many small varices in the anterior chest wall and mediastinum, especially on the left side (**A**, **B**)

Subsequent computed tomographic venography of the chest and neck revealed a thrombus obstructing the SVC around indwelling pacemaker leads (Fig. 1B). The image also showed many small varices in the anterior chest wall and mediastinum, especially on the left side, indicating the reconstruction of thoracic collaterals (Fig. 2A, B). However, considering that the patient still had thrombosis despite the original anticoagulant drug, he chose to continue rivaroxaban and up the dose to 20 mg daily. After 4 months of follow-up, the patient was aware of slight facial edema in the morning, but the symptoms of obstruction were significantly relieved. His symptoms had completely resolved nine months later.

## Discussion and conclusion

SVC syndrome refers to the obstruction of venous flow in the superior vena cavaIntrathoracic, malignancies are responsible for 60–85% of cases of SVC syndrome, and the occurrence of device-related SVC obstruction has increased and accounts for around 20–40% of overall cases [3]. Procedures performed on venous vasculature, resulting in a possible intimal injury or venous stenosis, provoked by transvenous leads, seem to be the most plausible explanation for the observed complication [4]. And what is striking, really remarkable, is when leads were serially evaluated with Doppler ultrasonography after initial placement, nearly 25% had associated thrombus, the majority of which occurred within 3 months [5]. In our case, although the patient had been taking anticoagulants for paroxysmal atrial fibrillation, he developed SVC syndrome after placement of a pacemaker for about 2 years. Thus it can be seen the patient's clotting was normal and no tumor was detected, but he was still at high risk for thrombosis.

Almost half of the SVC syndrome patients have relevant pulmonary embolism, which should prompt physicians to take positive therapy. Unfortunately, however, treatment options for device-related SVC obstruction are mainly supported by case series and anecdotal experience. Anticoagulation remains a common practice. The current practice is to start warfarin after 5 to 7 days of low-molecular-weight heparin. Other treatment options include lead extraction, thrombolysis, venoplasty,stenting, thrombectomy, and surgical grafts [6]. DOACs are now considered an important first-line therapy, and it has been reported that anticoagulation with Edoxaban 60 mg once daily could make complete resolution of the thrombosis after 3 months [4]. Although the etiology of pacemaker induced SVC syndrome is not identical to that of upper extremity deep vein thrombosis (UEDVT), it is important to note that the risk factors for UEDVT also include implantable pacemakers. The Swedish retrospective clinical study by Montiel et al. enrolled 55 patients treated with DOAC for 3–6 months because of UEDVT, the vast majority (84%) were treated with rivaroxaban, whereas 13% and 4% got apixaban and dabigatran, respectively., and found that DOAC can be used in the treatment of UEDVT patients with acceptable efficacy and safety [7]. The Italian multicenter retrospective study by Porfidia et al. also supports the concept that DOACs might be safe and efective for treating UEDTV [8]. Due to the similar pathophysiological mechanism, the research results and treatment experience of the two can learn from each other.

However, the choice of anticoagulant and duration of therapy remains controversial in the setting of SVC syndrome related to device leads. It is currently recommended to start 3 months of anticoagulant therapy, if symptoms persist, invasive venography should be performed to further characterize the source of occlusion. In a Canadian prospective multicenter study (CATHETER-2), 70 patients received rivaroxaban 15 mg twice daily for 3 weeks, followed by 20 mg once daily, in 51% of cases preceded by LMWH. This trial showed promise in treating central venous catheter (CVC)-UEDVT in cancer patients. However, raised concerns abput the risk of major bleeding and clinically relevant nonmajor bleeding, which occurred in 13% of patients during the 3-month follow-up [9]. This finding could be partly explained by the loading dose of rivaroxaban, since most of the events occurred in the first month. For our patient, considering the small amount of blood ooze in the left eye, we once again recommended the patient to switch to warfarin so that severe bleeding could be managed in time. However, the patient still required rivaroxaban, and we eventually increased the dose to 20 mg per day to achieve a therapeutic effect. Surprisingly, there were no significant abnormalities in coagulation function after increasing the dosage of anticoagulant drugs, no associated adverse events were observed. His symptoms of obstruction improved significantly after 4 months. Fortunately, the patient had complete resolved nine months later and has been taking adequate rivaroxaban anticoagulant.

**Fig. 3:**
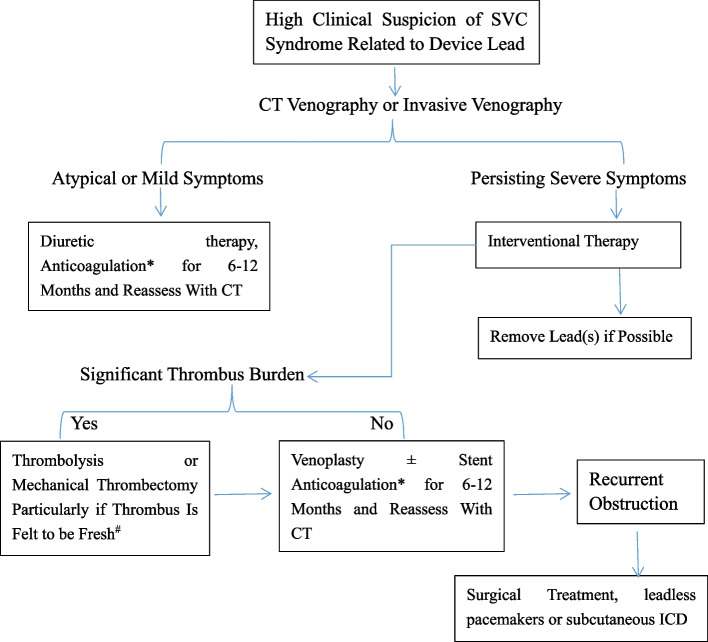
This figure shows an approach to the diagnosis and management of lead-associated superior vena cava (SVC) obstruction. ^#^ If thrombus is in the first 2–4 weeks following device implantation. *If leads are left in place, consider lifelong anticoagulation. CT: computed tomography; SVC: superior vena cava

For occlusions that are relatively acute or subacute (within weeks to months) with relatively fresh thrombus, debulking strategies, including the local administration of thrombolytics or the use of venous thrombus extraction devices, should be considered. If leads are reimplanted or left in place, lifelong anticoagulation is recommended [2]. Despite the DOACs being increasingly used in real-world experiences, randomized trials for the management of the pacemaker lead induced SVC syndrome is still lacking. For the management process of Lead-Associated SVC Obstruction, we can refer to Fig. 3.

Our case highlights the medical management of SVC syndrome with its early detection and the clear effect of the use of DOACs. This is a good option for patients who do not want to undergo interventional or surgical procedures.

## Data Availability

Not applicable.

## Abbreviations

SVC: Superior Vena Cava
DOACs: Direct Oral Anticoagulants
UEDVT: Upper Extremity Deep Vein Thrombosis
CT: Computed tomography
CVC: Central venous catheter

## References

[1] Rice TW, Rodriguez RM, Light RW (2006). The superior vena cava syndrome: clinical characteristics and evolving etiology. Medicine (Baltimore).

[2] Zimetbaum P, Carroll BJ, Locke AH, Secemsky E, Schermerhorn M (2022). Lead-Related Venous Obstruction in Patients With Implanted Cardiac Devices: JACC Review Topic of the Week. J AM COLL CARDIOL.

[3] Mir T, Uddin M, Shafi O, Qureshi W, Kaur J, Zghouzi M, Lohia P, Soubani A, Burket M, Sheikh M, Saydain G. Thrombotic superior vena cava syndrome: a national emergency department database study. J Thromb Thrombolysis. 2021;53(2):372–79. 10.1007/s11239-021-02548-7 34342784

[4] Mumoli N, Mazzone A, Evangelista I, Cei M, Colombo A (2021). Superior vena cava syndrome after pacemaker implantation treated with direct oral anticoagulation. Thromb J.

[5] van Rooden CJ, Molhoek SG, Rosendaal FR, Schalij MJ, Meinders AE, Huisman MV (2004). Incidence and risk factors of early venous thrombosis associated with permanent pacemaker leads. J Cardiovasc Electrophysiol.

[6] Kusumoto FM, Schoenfeld MH, Wilkoff BL, Berul CI, Birgersdotter-Green UM, Carrillo R, Cha YM, Clancy J, Deharo JC, Ellenbogen KA, Exner D, Hussein AA, Kennergren C, Krahn A, Lee R, Love CJ, Madden RA, Mazzetti HA, Moore JC, Parsonnet J, Patton KK, Rozner MA, Selzman KA, Shoda M, Srivathsan K, Strathmore NF, Swerdlow CD, Tompkins C, Wazni O (2017). 2017 HRS expert consensus statement on cardiovascular implantable electronic device lead management and extraction. Heart Rhythm.

[7] Montiel FS, Ghazvinian R, Gottsäter A, Elf J (2017). Treatment with direct oral anticoagulants in patients with upper extremity deep vein thrombosis. Thromb J.

[8] Porfidia A, Agostini F, Giarretta I, Tonello D, Pastori D, Pignatelli P, Santoliquido A, Sartori M, Lessiani G, Visonà A, Donadini MP, Pola R (2020). Upper extremity deep vein thrombosis treated with direct oral anticoagulants: a multi-center real world experience. J Thromb Thrombolysis.

[9] Davies GA, Lazo-Langner A, Gandara E, Rodger M, Tagalakis V, Louzada M, Corpuz R, Kovacs MJ (2018). A prospective study of Rivaroxaban for central venous catheter associated upper extremity deep vein thrombosis in cancer patients (Catheter 2). Thromb Res.

